# Personality traits and decision-making styles among obstetricians and gynecologists managing childbirth emergencies

**DOI:** 10.1038/s41598-023-32658-6

**Published:** 2023-04-05

**Authors:** Gabriel Raoust, Petri Kajonius, Stefan Hansson

**Affiliations:** 1 Division of Obstetrics and Gynecology, Department of Clinical Sciences Lund, Lund University, BMC C14, 221 84 Lund, Sweden; 2 Department of Psychology, Lund University, Box 213, 221 00 Lund, Sweden; 3 Women’s Health Clinic, Skåne University Hospital, Klinikgatan 12, 221 85 Lund, Sweden; 4 Women’s Health Clinic, Ystad Hospital, Kristianstadvägen 3A, 271 33 Ystad, Sweden

**Keywords:** Human behaviour, Psychology, Health care, Health occupations, Risk factors

## Abstract

The successful management of a childbirth emergency will be dependent on the decision-making of involved obstetricians and gynecologists. Individual differences in decision-making may be explained through personality traits. The objectives of the present study were (I) to describe personality trait levels of obstetricians and gynecologists and (II) to examine the relationship between obstetricians’ and gynecologists’ personality traits and decision-making styles (Individual, Team and Flow) in childbirth emergencies; also controlling for cognitive ability (ICAR-3), age, sex and years of clinical experience. Obstetricians and gynecologists, members of the Swedish Society for Obstetrics and Gynecology (N = 472) responded to an online questionnaire that included a simplified version of the Five Factor Model of personality (IPIP-NEO), and 15 questions concerning childbirth emergencies based on a model of decision-making styles (Individual, Team and Flow). The data was analyzed using Pearson’s correlation analysis and multiple linear regression. Swedish obstetricians and gynecologists scored (*P* < 0.001) lower on Neuroticism (Cohen’s d = − 1.09) and higher on Extraversion (d = 0.79), Agreeableness (d = 1.04) and Conscientiousness (d = 0.97) compared to the general population. The most important trait was Neuroticism, which correlated with the decision-making styles Individual (r = − 0.28) and Team (r = 0.15), while for example Openness only trivially correlated with Flow. Multiple linear regression showed that personality traits with covariates explained up to 18% of decision-making styles. Obstetricians and gynecologists have notably more distinct personality levels than the general population, and their personality traits relate to decision-making in childbirth emergencies. The assessment of medical errors in childbirth emergencies and prevention through individualized training should take account of these findings.

## Introduction

Giving birth is relatively safe in the wealthiest parts of the world^1^. However, there are puzzling variations in delivery outcomes and intervention rates between different high-income countries, and between maternity units within the same country, regardless of universal coverage and standardized care^2,3^. Decisions and actions affecting childbirth, particularly during emergencies, often emerge from the coordinated efforts of the obstetric personnel^4,5^. Nevertheless, the resilience and success of the group will be dependent on the individuals’ competence, characteristics, and adaptability^4,6,7^. Such individual differences and the resulting variations in response to similar emergency situations can be seen as the result of personality traits^8,9^. Scientifically, personality traits are organized according to the Five Factor Model (FFM; Neuroticism (e.g., emotional instability, anxiety and pessimism), Extraversion (e.g., sociability and assertiveness), Openness (e.g., intellect and curiosity), Agreeableness (e.g., compassion and civility) and Conscientiousness (e.g., responsibility and achievement))^10,11^. The replicable framework of the FFM has led to a substantial research literature linking personality to various individual, interpersonal, and social-institutional outcomes^8,9,11,12^. For physicians working in obstetric care, only a few studies have discussed the impact of personality on the choice of this specialty, on physicians’ responses to emergency stress, on teamwork, or obstetrical outcomes^13–17^. To the best of our knowledge no previous research has explored the personality of obstetricians and gynecologists in relationship to decision-making during childbirth emergencies. The aims of this study were: (I) to describe the personality trait levels of obstetricians and gynecologists and (II) to examine the relationship between obstetricians’ and gynecologists’ personality traits and decision-making styles in childbirth emergencies.

## Methods

### Ethics declaration

The study was approved by the regional ethics review board (Lund University, permit number LU 2018/198). Informed consent was obtained from the study participants. Participation could be terminated at any time. All methods were carried out in accordance with relevant guidelines and regulations.

### Instruments

See Supplementary Questionnaire S1. Personality traits were assessed using a shorter 30-item version of the FFM standardized psychometric pool of items (IPIP-NEO; See http://ipip.ori.org)^18^. The measure consists of 6 items for each of the five factors on a Likert-scale 1 (not at all, almost never) to 5 (very much, almost always). Decision-making was assessed using 15 questions reflecting three decision-making styles: an individual-centered, a team-based and a flow-oriented (See Table 1 for items for each decision-making style). These items were based on the results from a previous study^19^ and are also supported by evidence from the literature^16,20–26^. The same Likert-scale was used for measure. Control variables, acting as covariates in our analyses were cognitive ability, age, sex and years of clinical experience. Cognitive ability was assessed using 3 spatial items in the form of three cube rotations from the International Cognitive Ability Resource (ICAR-3; See https://icar-project.com/)^27^.

**Table 1:** Three decision-making styles during childbirth emergencies.

Decision-making styles	Items (During emergencies…)
*Individual-centered* an agent-centered, rational decision-making style characterized by a thorough search for and logical evaluation of alternatives ^19,28^	… the responsibility rests with me … I take in information, process and give directives … guidelines are important … structure creates a sense of safety … there are right and wrong decisions
*Team-based* a dependent decision-making style characterized by a search for advice and direction ^19,28^	… my focus is on the birthing woman and her partner … it’s nice to have a sparring partner … we help each other out in the team … different team members’ contributions are important … I think of the consequences for the birthing woman
*Flow-oriented* an intuitive decision-making style characterized by reliance and hunches and feelings ^19,28^. Flow refers to a relatively uncommon state of mind in which the person performing an activity is fully and intuitively immersed in a feeling of energized focus and enjoyment in the process of that activity ^24^	… I trust my intuition … I don’t always know what’s right … I sometimes need to improvise … The outcome is beyond my control but it’s important that everyone does her/his best … I trust the process/higher powers

Items refer to question 23.1–23.15 in the questionnaire.

### Sample and procedure

The questionnaire was sent out to all Swedish obstetricians and gynecologists, members of the Swedish Society for Obstetrics and Gynecology during 2 months in 2020. According to the organization’s 2019 annual report, it comprised 2180 members, including 480 retired physicians. Of the initial 513 responses forty-one were excluded, due to erratic or duplicate responses. A sample of N = 472 (79% women, M_Age_ = 46.4 years, 28–90 years) which we call Ob&Gyn was used for all analyses. All sample characteristics are shown in Table 2. The number of respondents from each of the six health care districts in Sweden was proportional to the number of its inhabitants and births (Supplementary Fig. S2). The personality trait levels in Ob&Gyn were also compared with the general population. A sample from the general Swedish population (N = 1943) was used as reference group. This sample was collected via an anonymous voluntary personality-testing website, using the same items (IPIP-NEO) as the present study^18^. The number of women and men in the reference sample was approximately equal (48% women, M_Age_ = 29.6 years, 19–66 years).

**Table 2:** Descriptive characteristics of the Ob&Gyn sample (N = 472).

	M	SD	Skewness	Kurtosis	Cronbach’s α	Sex differences^a^ (Cohen’s d)	Differences with reference sample (N = 1943)^a^ (Cohen’s d)
Decision-making styles							
Individual	4.37	0.46	− 0.49	0.10	.67	NS	–
Team	4.67	0.51	− 1.27	2.20	.62	− 0.45	–
Flow	3.20	0.63	0.16	− 0.46	.60	NS	–
Personality traits							
Neuroticism	1.97	0.60	0.66	0.23	.82	− 0.45	− 1.09
Extraversion	3.81	0.65	− 0.37	− 0.04	.78	NS	0.79
Openness	3.73	0.67	− 0.49	0.11	.73	NS	NS
Agreeableness	4.27	0.48	− 0.47	− 0.14	.62	− 0.56	1.04
Conscientiousness	4.12	0.54	− 0.66	0.45	.73	− 0.30 (*p* = 0.014)	0.97
Covariates							
Cognitive ability	1.06	1.13	0.59	− 1.11	–	NS	–
Age	46.4	12.3	0.66	− 0.38	–	0.68	1.54
Clinical experience	15.5	12.1	0.73	− 0.43	–	0.61	–

^a^Welch’s t-test was used, Levene’s test being significant (*p* < 0.05); all differences are significant on the *p* < 0.001 level, except when specified otherwise.

### Statistical analyses

If Levene's test was significant (*P* < 0.05), suggesting a violation of the assumption of equal variances, Welch’s t-test instead of Student’s t-test, was used for comparisons. Reliability analyses were conducted with Cronbach’s alpha. Zero-order Pearson’s correlations were used to assess relationships between personality traits and decision-making styles. Multiple linear regression analysis was performed for each dependent decision-making style Individual, Team and Flow with all five personality traits, together with covariates as independent variables. The following cut-off values were used to assess the strength of a correlation or regression estimate: r ≥ 0.30, a strong correlation and r ≤ 0.20 a weak correlation^29^. A preparatory power calculation aiming to find effects larger than r > 0.15 (α = 0.01, 75% power) indicated a sample size of N = 465^30^. All statistical analyses were performed using the open source program *Jamovi, v. 2.3.21*.

## Results

In order to test the first aim of the study, the levels of personality traits in obstetricians and gynecologists were assessed and are described in Table 2. Agreeableness showed the highest levels, while Neuroticism the lowest. Average length of clinical experience was 15.5 years. Women scored higher than men in Neuroticism (d = 0.45), Agreeableness (d = 0.55) and Conscientiousness (d = 0.31). To further understand personality trait levels with obstetricians and gynecologists, the results were compared to a Swedish reference group (N = 1943). A Welch’s t-test showed very large differences with obstetricians and gynecologists having lower scores in Neuroticism (d = − 1.09), higher in Extraversion (d = 0.79), Agreeableness (d = 1.04) and Conscientiousness (d = 0.97). The second aim was to evaluate correlations between the five personality traits and three decision-making styles (Table 3). The results showed that Neuroticism was negatively correlated with Individual decision-making, and slightly positive with Team decision-making. Overall, Extraversion, Openness, Agreeableness were positively correlated with both Individual and Team decision-making, as well as each other. The correlations between decision-making styles were overall small.

**Table 3:** Correlations between personality traits and decision-making styles.

	1	2	3	4	5	6	7	8
1. Neuroticism		− 0.32			− 0.39	− 0.38	0.21	
2. Extraversion	− 0.26		0.24	0.33	0.30	0.19	0.19	
3. Openness		0.18		0.27		0.24	0.24	0.17
4. Agreeableness		0.23	0.18		0.33	0.31	0.29	
5. Conscientiousness	− 0.30	0.23		0.22		0.24		
6. Individual	− 0.28	0.14	0.17	0.20	0.17		0.14	0.19
7. Team	0.15	0.13	0.16	0.18		0.09		0.25
8. Flow			0.11			0.12	0.15	

Above the diagonal are disattenuated correlations, controlled for unreliability (Cronbach’s α). All correlations above 0.15 are significant on the *p* < 0.01 level.

Two step multiple linear regressions, one for each decision-making style, and with covariates were also conducted (Table 4). After controlling for age, sex, and clinical experience, Neuroticism was still significantly (*P* < 0.001) negatively related to the Individual decision-making style. Women and older age were positively related to Team; while the more years of clinical experience the less decision-making was based on Team. Cognitive ability did not show any relationships to decision-making style.

**Table 4:** Regression models: the effects of personality traits on the decision-making styles.

	Individual		Team		Flow	
	R^2^ = 0.13 F(5,433) = 13.20 *p* < 0.001	R^2^ = 0.18 F(9,419) = 10.20 *p* < 0.001	R^2^ = 0.09 F(5,435) = 8.74 *p* < 0.001	R^2^ = 0.15 F(9,421) = 7.93 *p* < 0.001	R^2^ = 0.03 F(5,430) = 3.01 *p* = 0.011	R^2^ = 0.05 F(9,417) = 2.20 *p* = 0.021
	β	β	β	β	β	β
Neuroticism	− 0.24	− 0.17	0.20	0.12 (*p* = 0.025)	0.06	0.07
Extraversion	− 0.01	0.00	0.11 (*p* = 0.023)	0.10 (*p* = 0.041)	− 0.06	− 0.06
Openness	0.11 (*p* = 0.013)	0.09	0.13 (*p* = 0.005)	0.16	0.12 (*p* = 0.012)	0.11 (*p* = 0.021)
Agreeableness	0.16	0.15 (*p* = 0.002)	0.13 (*p* = 0.007)	0.11 (*p* = 0.021)	0.08	0.07
Conscientiousness	0.08	0.07	0.03	0.02	− 0.07	− 0.08
Cognitive ability		− 0.01		0.04		− 0.08
Age		0.02		0.31 (*p* = 0.019)		− 0.03
Sex		0.05		0.26 (*p* = 0.032)		0.16 (NS)
Clinical experience		0.22 (NS)		− 0.46		0.11

*SE* Standard error (0.00–0.08), β = standardized estimate. All estimates above 0.15 are significant *p* < 0.001, except when specified otherwise. Men = 0, Women = 1.

## Discussion

The present study showed that Swedish obstetricians and gynecologists have notably different personality trait levels on four out of five trait factors (including lower Neuroticism and higher Extraversion, Agreeableness, and Conscientiousness) compared to the general Swedish population. Such differences are not unusual to find in the literature regarding linkage between personality and occupational choices^12^ or academic disciplines^31^. It may be that Swedish obstetrics oriented physicians choose and thrive in what best suits their personality trait levels. Childbirth emergencies are potentially high stake situations that can quickly escalate to crisis^32^. It is in this setting that physicians’ personalities are put to the test^19^. A lower level of Neuroticism would be an advantage for dealing with stress and uncertainty in such moments^33,34^. Similarly, a higher level of Extraversion, being comfortable in taking lead as well as enjoying a team challenge, would also be an advantage^33^. Higher levels of Agreeableness, especially combined with a high level of Conscientiousness, would be favorable in situations that naturally involve following procedure and check lists while interacting with team members, particularly during emergencies^35^. Further research into what motivates physicians to choose and stay in the specialty of obstetrics and gynecology would be valuable. The present study also showed that personality can predict which decision-style that is preferred. Low Neuroticism, which stands for emotional stability, was the most important trait, showing non-trivial effects even after controlling for cognitive ability, age, sex, and clinical experience. Neuroticism is characterized by anxiety and vulnerability to stress and showed a negative relationship with Individual decision-making and a positive with Team decision-making. A more positive aspect of Neuroticism has been shown to translate into caution and dialogue with peers^36,37^. Women are also known to have higher Neuroticism than men and to take fewer risks^38^. Also, Neuroticism is known to be tempered with age^9^. Concerning clinical experience, the results showed a negative relationship to the Team-style, which suggest that the less experienced physicians are, the more they turn to a Team-based decision-making. Today there is a consensus that teamwork is not only an essential normal part of daily work, but also the key to solving many complex clinical problems^22,23,39–41^. Also, Openness showed a relationship with the Team-style, working with others, as well as with the Flow-style, which also is in line with the nature of Openness, a trait characterized by listening in to others, as well as having the liking to be immersed in an intuitive kind of decision-making. Overall, the present study show indications that the Team-style of decision-making is facilitated by the traits of Neuroticism, Agreeableness, and Openness, especially by older women, with fewer years of clinical experience. The results give an enhanced comprehension of the various factors, including personality traits, affecting the physicians' decision-making processes during childbirth. An individual-centered or a team-based approach may be appropriate depending on the individual. This may be surprisingly relevant even in a highly organized and protocol-driven environment such as emergency obstetrics. In the future, it may be of value to incorporate clinical experience and personality when selecting candidates for the Obstetrics and Gynecology specialty, trainee education, and the assessment of medical errors related to childbirth.

## Limitations

There are several limitations to the present study. Concerning the first aim, to describe the personality trait levels of obstetricians and gynecologists, even though using the same personality instrument (IPIP-NEO) the reference sample used for comparison was much younger and from various professional backgrounds. It is precarious to draw strong conclusions from these analyses; however, the effect sizes were very large. One standard deviation of difference in for instance Neuroticism, Agreeableness, and Conscientiousness converts into 85% of all the Ob&Gyn sample practitioners having trait levels above the reference sample mean. Similarly, the reference sample unlike the Ob&Gyn sample was evenly distributed in men and women, which likely could explain part of the difference, especially in Neuroticism and Agreeableness. Furthermore, it may be that the physicians who chose to take the time and effort to answer the questionnaire were characterized by higher Agreeableness, and Conscientiousness thus inflating the differences by selection bias.

Nevertheless, this is likely the first time this particular profession has been evaluated based on the Five Factor Model of personality. Concerning the second aim, predicting decision-making based on personality traits for childbirth emergencies in real time is a complex phenomenon and conceptualization into styles, as attempted for the purpose of this study may be insufficient^4,32,42^. Arguably, the somewhat low reliabilities (Cronbach’s alphas.60–.67) support this notion. It could also be debated whether controlling for cognitive ability, age, sex, and clinical experience was meaningful, seeing how the study aim was to focus on personality traits in relation to decision-making. Personality traits do differ between age groups, between sexes, and for those who stay in the profession long enough to gain clinical experience, which now are obscured within the covariates. Due to this, along with the modest reliabilities with decision-making styles, we also presented disattenuated correlations in Table 3. Common method variance, which is the tendency of participants to somewhat agree with items, might also have confounded the results somewhat^43^. This effect is likely only in parity with the smallest correlation (r = 0.09), found between decision-making styles. Further explorations regarding the role of personality traits in decision-making styles during childbirth emergencies is warranted.

## Supplementary Information

Supplementary Information 1.

Supplementary Information 2.

## Data Availability

The dataset generated and analyzed during the current study is available upon request to The Swedish National Data Service, and for research purposes only, https://snd.gu.se/en/catalogue/study/2022-57.
