# Supplementary figures and images for: Personality traits and decision-making styles among obstetricians and gynecologists managing childbirth emergencies
Source: Sci Rep. 2023 Apr 5;13:5607. doi: 10.1038/s41598-023-32658-6 (PMC10076329; doi:10.1038/s41598-023-32658-6)

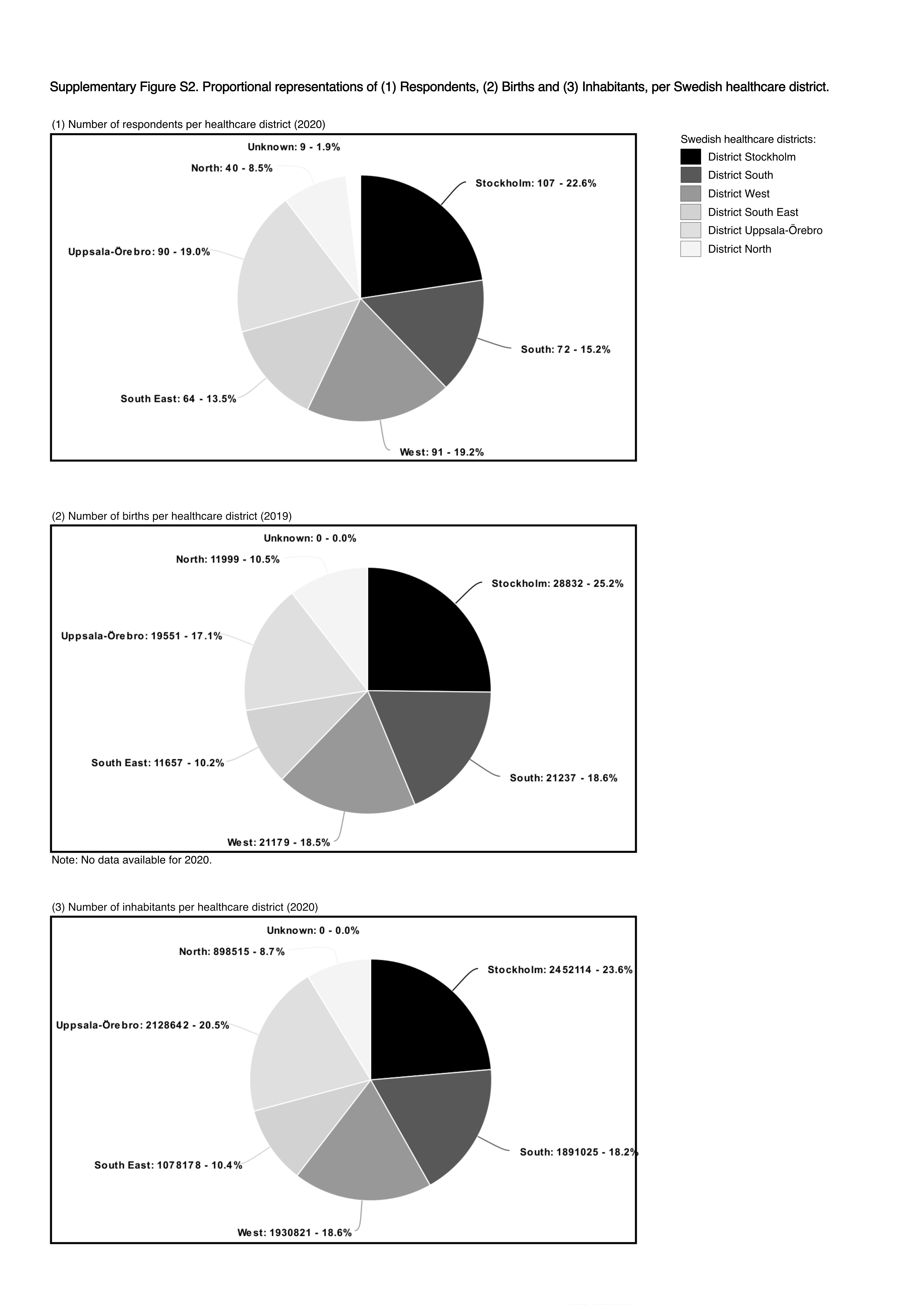

Supplement: Supplementary file 1 — Supplementary Information 1. [file 41598_2023_32658_MOESM1_ESM.jpg]
