# Supplementary material for: Personality traits and decision-making styles among obstetricians and gynecologists managing childbirth emergencies
Source: Sci Rep. 2023 Apr 5;13:5607. doi: 10.1038/s41598-023-32658-6 (PMC10076329; doi:10.1038/s41598-023-32658-6)
Supplement: Supplementary file 2 — Supplementary Information 2. [file 41598_2023_32658_MOESM2_ESM.docx]

Preferences in Decision Making During Obstetric emergencies

Swedish maternity and intrapartum care is designed in a way to ensure equality of services throughout the country. Despite this, regional differences still persist regarding the frequency of a number of perinatal outcomes such as caesarean sections, lacerations, etc. A couple of American studies have suggested that physicians' personality traits are of importance for outcomes in intrapartum care. However, it is a question of whether personality can have similar effects in Sweden, where healthcare differs significantly from that in the US. The purpose of this study is to analyse preferences in decision-making and personality traits among Swedish gynecologists and obstetricians.

We wonder if you, as a member of SFOG, would consider participating in this study. It takes about 15-20 minutes to complete the survey. The research results will be used as part of the ongoing effort to improve Swedish maternity care. The intention is above all, to increase patient safety. Your contribution is valuable and we hope that it can feel motivating enough to participate.

By completing the form and by clicking on 'submit' on the last page, you certify that you have voluntarily consented to participate, in accordance with the latest GDPR (Personal Data Act). You can withdraw your participation at any time. All data is anonymous and encrypted, and no third party will have access to how you specifically answered a question. The quality of the answers is based on sincerity, generosity, and integrity.

Please note that there are no right or wrong answers. See your participation as a serious opportunity to contribute to understanding more about an individual's unique profile. The methods used in the survey are based on thousands of hours of scientific studies and underlying algorithms.

You will have to decide on a number of statements throughout six pages, requiring a bit of focused time. Our best advice is to have fun while filling out the form, keep a steady pace, and above all, trust your first, intuitive reaction - sincerity is rewarded by the analysis formulas.

Lets start.

General Information

1. Tell us if you are a woman or a man (for statistics)

Man

Woman

I don’t identify as either woman or man

1. Tell us your age (for statistics)

1. Tell us where you work, city/region (for statistics)
2. To what extent are you clinically active?

20% or less

Between 20 and 50%

50%

Between 50 and 100%

100%

1. Tell us how many years you’ve worked with obstetrics and gynecology

Thank you for answering this first part.

Obstetrics or gynecology?

1. Which do you prefer?

Strongly Disagree Neither agree Agree Strongly agree

disagree nor disagree

Obstetrics

Gynecology

How well do the following statements relate to you? Answer preferably quickly, honestly and intuitively.

1. I like obstetrics because…

Strongly Disagree Neither agree Agree Strongly agree

disagree nor disagree

Patient contact is usually

quite limited.

You make quick, crucial

decisions.

You get to be part of

very special moments.

1. I like gynecology because...

Strongly Disagree Neither agree Agree Strongly agree

disagree nor disagree

I help women.

Sick patients get well

often.

Surgery is particularly

stimulating.

1. I would have NOT liked to only work with obstetrics because...

Strongly Disagree Neither agree Agree Strongly agree

disagree nor disagree

There is too much

uncertainty with/around

pregnancy and childbirth.

I feel uncomfortable with

the responsibility during

childbirth.

1. I would have NOT liked to only work with gynecology because...

Strongly Disagree Neither agree Agree Strongly agree

disagree nor disagree

I would get bored from

that type of interaction with

patients.

I can’t manage to stay

concentrated for too

long during surgery.

1. Briefly describe what motivates your preference.

We appreciate that you’re still interested to continue the survey.

Personality traits

How well do the following statements relate to you? Answer preferably quickly, honestly and intuitively.

12. Strongly Disagree Neither agree Agree Strongly agree

disagree nor disagree

Get easily stressed

Often feel anxious

Find it sometimes

difficult to deal with

things

Get easily panicked

Often feel down

Fear the worst

13. Strongly Disagree Neither agree Agree Strongly agree

disagree nor disagree

Feel comfortable together

with others

Have an easy time

making friends

Avoids people

Talk to a lot of different

people during a party

Do a lot of fun things

Dislike crowded places

14. Strongly Disagree Neither agree Agree Strongly agree

disagree nor disagree

Believe in the importance

of art

See beauty where others

seldom do

Have strong imagination

Avoid philosophical

discussions

Have difficulty understanding

abstract theories

Dislike going to museums

15. Strongly Disagree Neither agree Agree Strongly agree

disagree nor disagree

Get emotionally affected

by others

Am indifferent to other’s

feelings

Use others for my own

needs

Do not give time for others

Am uninterested in other

people’s problems

Feel sympathy for those

that have it worse than me

16. Strongly Disagree Neither agree Agree Strongly agree

disagree nor disagree

Waste my time

Follow through with

my plans

Work hard

Always complete my

tasks

Know how to get things

done

Just do as much as

I need to

Good job! You’ve done more than half.

Spatial understanding

Which of the following alternatives is a rotated version of cube X?

Your answers are important, please keep focus. And don't use Google search.

17.


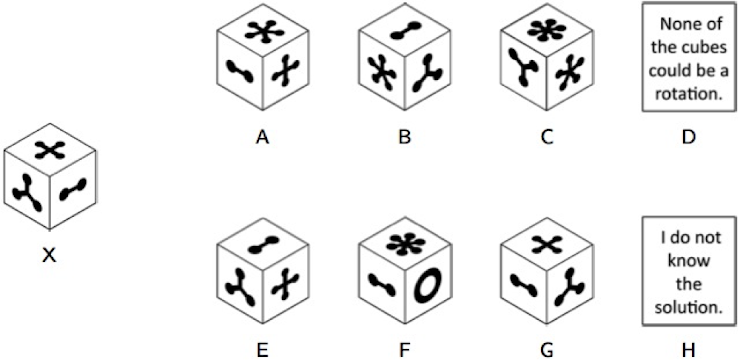


A B C D E F G H

18.


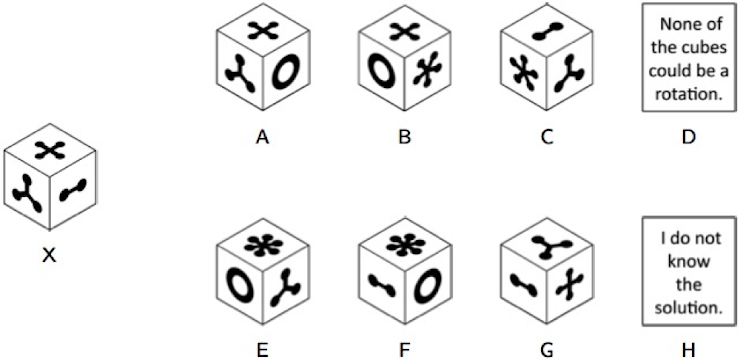


A B C D E F G H

19.


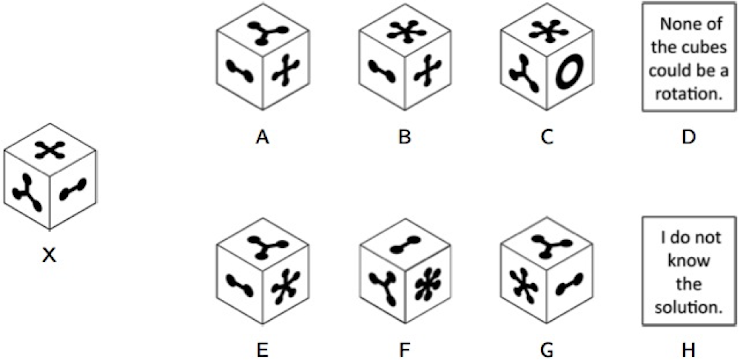


A B C D E F G H

Good job! Only one part left.

This last part is a little longer, but don’t give up. Your answers are important, please stay focused.

Obstetric emergencies

20. Obstetric emergency 1.

You work as a young trainee in a medium-sized maternity unit with about 2,500 births per year. It’s 01:30 at night. Your senior is 30 minutes away. You’ve taken care of a few gynecological emergencies, and have worked non-stop since the beginning of your shift at 16:30. You recon it’s finally settling down and think of going to your room in the hope of getting a couple of hours sleep. But, you just want to check on the delivery suites one extra time. On your way there, the phone rings. It’s a somewhat anxious voice at the other end: “the midwife wants you to come, there’s a bad tracing”. She hangs up before you even get the chance to ask anything. To your general state of anxiety and fatigue now adds a layer of annoyance. Finally inside the right suite, your gaze almost automatically turns to the CTG. The woman, who is giving birth to her second child, is kneeling in bed and leaning against the headboard. The partner is super-peppery. The midwife says: “she’s fully dilated and the head is pretty far down. I just want you to check the CTG”. You see a pathological tracing with repetitive complicated variable decelerations for just over 7 minutes. Variability is still good but the baseline rate seems to fall slowly.

How well do the following statements relate to you? Give an honest answer of what you would have done.

Strongly Disagree Neither agree Agree Strongly agree

disagree nor disagree

CTG is pathological.

I tell the midwife that we

should immediately

deliver with a ventouse

(ie vacuum extractor).

The patient should shift

position to either her

side or her back.

I greet the patient and

her partner. I listen to the

midwife’s report, and take

peek at the CTG at the

same time.

Together, we get the patient

to shift position. The midwife

wants to try an let the patient

push a little more, which I

think is reasonable.

CTG seems to have been

normal earlier on, we

give it a chance but I stay

in the suite.

I take a lactate sample while

the woman is standing on her

knees, when the head is

coming down, just to be

sure.

21. Obstetric emergency 2.

You work as a young trainee in a medium-sized maternity unit with about 2,500 births per year. It’s 01:30 AM. Your senior is 30 minutes away. You’ve taken care of a few gynecological emergencies, and have worked non-stop since the beginning of your shift at 16:30. You recon it’s finally settling down and think of going to your room in the hope of getting a couple of hours sleep. But, you just want to check on the delivery suites one extra time. On your way there, the phone rings. An assistant nurse, who sounds a bit blasé, asks for “help on the third. The midwife wants you to come and help out with an instrumental delivery. The mom’s too tired”. When you get to examine the patient, you sense that there is plenty of space but that the head might be a bit too high up. The contractions are strong and painful but short-lived. The woman’s giving birth to her first child and is completely exhausted. The CTG is normal with repetitive uncomplicated decelerations. You call for your senior but he does not answer the phone.

How well do the following statements relate to you? Give an honest answer of what you would have done.

Strongly Disagree Neither agree Agree Strongly agree

disagree nor disagree

The head is probably too

far up, patient is tired,

and it’s late: “probably best

to go for a c-section”.

I tell the midwife that the

patient should stand up

for an hour, after which

a new assessment should

be made, in the hope that

a ventouse or forceps can

be applied.

I hear with the midwife

what she thinks, and

I suggest: “we should

increase the oxytocin,

and let the patient try

to push”.

The assistant nurse says

that the patient has been

lying most of the time, and

that she is scared. After a

brief discussion outside the

suite, the midwife and I

suggest a plan for the

patient and her partner.

After a thorough examination

it feels as if it should work with

a ventouse. I tell the couple

that “it’s going to be fine”.

The midwife and I have

talked with each other, we

believe that there’s a fair

chance she can give birth

vaginally. While in the suite,

I ask the midwife to increase

the oxytocin, and ask for help

in hyperflexion of the hips.

22. Obstetric emergency 3.

You work as a young trainee in a medium-sized maternity unit with about 2,500 births per year. It’s 01:30 AM. Your senior is 30 minutes away. You’ve taken care of a few gynecological emergencies, and have worked non-stop since the beginning of your shift at 16:30. You recon it’s finally settling down and think of going to your room in the hope of getting a couple of hours sleep. But, you just want to check on the delivery suites one extra time. On your way there, the phone rings. A panicky voice that you barely recognize says: “you need to come now, it's room 2. The child is stuck!” You immediately think of a shoulder dystocia, and in the back of your mind “god I do not hope so”… Barely a couple of minutes later you enter the delivery suite, short of breath. The midwives’ desperate gazes, and what you feel is as a mixture of fear and anxiety in the partner’s face meet you. The patient pushes with all she’s got. One of the midwives says: “I can’ t get hold of the posterior shoulder, it’s been 4 minutes now”.

How well do the following statements relate to you? Give an honest answer of what you would have done.

Strongly Disagree Neither agree Agree Strongly agree

disagree nor disagree

I take control of the situation

and start the HELPERR

sequence, according to

guidelines.

It is my job to solve the

situation.

I first listen to what the

midwife has to say.

The midwife suggests to

use some nitroglycerine

spray, and that we try

HELPERR again, but

that I stand between the

patients legs.

I ask for permission to

get access, and see if

I can help the baby out.

I finally break the baby’s

collar bone,

Since nothing else

seems to work.

23. During obstetric emergencies…

Please respond to the following statements. Your answers are important. Be honest, nobody is here to judge you.

Strongly Disagree Neither agree Agree Strongly agree

disagree nor disagree

the responsibility rests with me.

I take in information, process and

give directives.

guidelines are important.

structure creates a sense of

safety.

there are right and wrong

decisions.

my focus is on the birthing

woman and her partner.

it’s nice to have a sparring

partner.

we help each other out in the

team.

the different team members’

contributions are important.

I think of the consequences

for the birthing woman.

I trust my intuition.

I don’t always know what’s

right.

I sometimes need to improvise.

the outcome is beyond my

control but it’s important that

everyone does her/his best.

I trust the process/higher powers.

24. Final feedback.

Strongly Disagree Neither agree Agree Strongly

disagree nor disagree agree

This survey was

important.

The questions were

relevant.

It was difficult to

answer.

It was rewarding for

me.

Thank you for your contribution and effort! We trust that you had a good time. No one knows you better than yourself. Just click ‘submit’ to finish and send us your answers.
